# Remaining Question: Does Exercise Improve Healing of Diabetic Foot Ulcers?

**DOI:** 10.1177/15347346211063701

**Published:** 2021-12-08

**Authors:** Magali Brousseau-Foley, Virginie Blanchette

**Affiliations:** 1 14847 Université du Québec à Trois-Rivières, Trois-Rivières, Canada; 2 Trois-Rivières Family Medicine University Clinic, Trois-Rivières, Canada

**Keywords:** Diabetic foot, Foot ulcer, Exercise, Wound healing, Systematic review

## Abstract

Even though it is reasonable to think that exercise is beneficial to diabetic foot ulcer healing, there are currently no exercise recommendations for this population. A systematic review published recently attempted to answer this question. However, because of both the scarce and heterogenous literature on the subject identified by the selected study design and the chosen quality appraisal tool that is too permissive and overestimates the treatment effects, no clinical recommendations can be drawn from this review. We advocate for research on this topic in order to obtain more direct evidence that exercise benefits wound healing, and to close the persistent gap of knowledge regarding the impact of exercise on diabetic foot ulcer wound healing.

Dear editors,

A recent systematic review has been published to answer the question “Does exercise improve healing of diabetic foot ulcers?.^1^ We would like to underline how relevant we believe this emerging topic is, and make some contributions.

As our research team recently completed a comprehensive review on the scope of the literature regarding physical activity participation in individuals with an active diabetic foot ulcer (DFU),^2^ we were surprised by Tran and Haley's conclusions based on the few articles included in their review.^1^ Indeed, only three articles met inclusion criteria, totalizing 139 participants from which 71 were allocated to three very different exercise regimens, all considered non-weight-bearing, with generally low participants’ adherence for the two interventions that measured this outcome, as the authors adequately acknowledge. They mention a certain amount of wound healing but it appears from the data that this is not generalized to all studies and that no attempt was made to correlate wound healing with the type or volume of exercise performed. Also, they state that there were no adverse consequences of exercise, but as another team of researchers that published a previous systematic review on the topic critiqued, this is often ill-defined and underreported as it is the case in the included studies.^3^ Based on the studies included by Tran and Haley, they conclude that there is insufficient evidence to support exercise to improve DFUs healing, but then they encourage exercise as part of the management plan for treatment of DFUs, which seems based solely on indirect evidence from *in vitro* studies or studies on populations without an active DFU. Systematic review authors always need to be cautious when expressing conclusions, and this case illustrates this well. Their recommendations are not adequately supported by evidence, and could even be detrimental when exercise is weight-bearing as concluded in another recent study.^4^

We were additionally perplexed by the choice of the Physiotherapy Evidence Database scale (PEDro scale) to assess the quality of the included studies. Compared to the Cochrane Risk of Bias tool (Cochrane RoB tool), a meta-epidemiological study found that while it was widely used in the literature, the PEDro scale deemed of adequate quality did not meet the accepted quality standards and overestimated treatment effects. ^5^ This was considered clinically relevant, and as the use of the PEDro scale could have a direct impact on the clinical recommendations and decisions, the use of the Cochrane RoB tool rather than the PEDro scale was advocated. To illustrate this, we provide in Figure 1 a quality assessment using the Cochrane RoB 2 tool, the 2019 updated version of the Cochrane RoB tool, of the studies included in the systematic review by Tran and Haley.^1^

**Figure 1.**
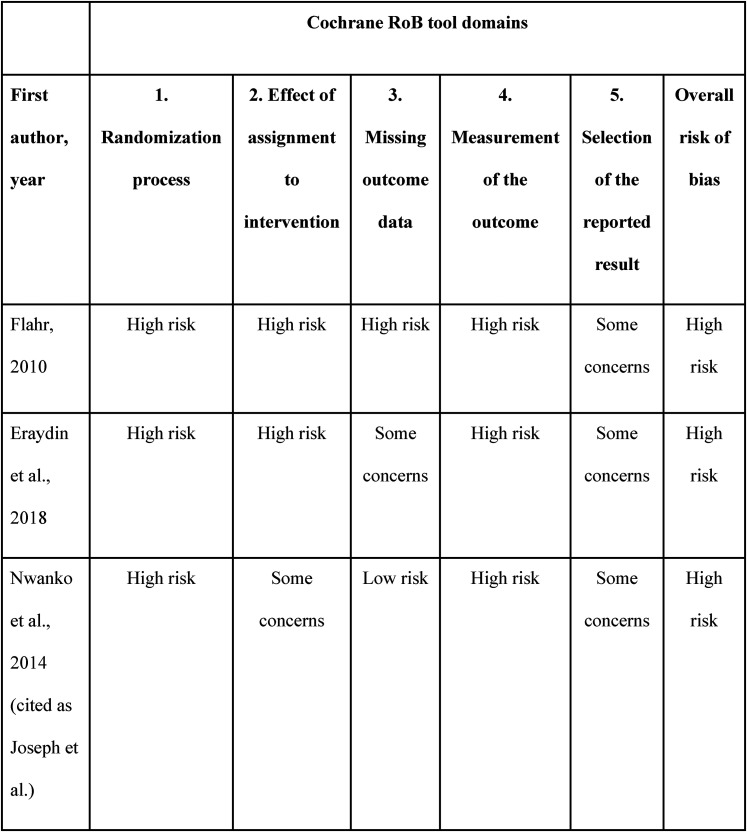
Risk of bias assessment using cochrane RoB 2 tool for tran and haley's review included studies.

Even though we understand that the chosen study design requires a narrow scope of research, it appears that the paucity of available data and their limited quality might have been better served by a more inclusive design. Moreover, a group recently published a position statement providing patients with recommendations based on the best available evidence and expert opinions on weight-bearing activity (which can include exercises) post DFU healing, and to avoid recurrence. ^6^ Their work supports limiting activity during DFU healing as much as possible. Finally, in the light of the available evidence, we believe a more nuanced conclusion would have reflected more adequately the persistent gap of knowledge regarding the impact of exercise on DFUs’ healing. A pragmatic clinical trial assessing the dose-response impact of selected exercises in individuals with an active DFU on wound healing parameters, adverse events, and global health outcomes is required to demonstrate how exercise actually benefits wound healing.

## Abbreviations

Cochrane RoB tool: Cochrane Risk of Bias tool
DFU: Diabetic Foot Ulcer
PEDro scale: Physiotherapy Evidence Database scale
